# Development and Evaluation of the Antibacterial Properties of an Experimental Herbal Gel Against Cariogenic Bacteria

**DOI:** 10.1002/cre2.70076

**Published:** 2025-01-22

**Authors:** Marco Sánchez‐Tito, Lidia Yileng Tay, Francisco Zea‐Gamboa, Raúl Cartagena‐Cutipa, Alysson Flores‐Gómez, Bruno Spigno‐Paco, Brando Raul Mendoza Salinas, Jose Elias Zuñiga Calcina, Ingrit Elida Collantes Díaz

**Affiliations:** ^1^ School of Dentistry, Faculty of Health Science Private University of Tacna Tacna Peru; ^2^ Faculty of Stomatology Cayetano Heredia Peruvian University Lima Peru; ^3^ Faculty of Chemistry and Textile Engineering National Engineering University Lima Peru; ^4^ Faculty of Biology National University of San Agustin Arequipa Peru

**Keywords:** antibacterial, caries, essential oil, medicinal plants

## Abstract

**Background:**

Recently, products with antibacterial properties derived from medicinal plants have increased as an alternative to conventional drugs. Thus, this study aimed to formulate and evaluate the antibacterial activity of an experimental gel based on *Grindelia tarapacana* essential oil in a bacterial consortium.

**Material and Methods:**

The composition of the essential oil (EO) was determined using gas chromatography‐mass spectrometry (GC‐MS). The antibacterial activity of the EO against *Streptococcus mutans* ATCC 25175, *Streptococcus sanguinis* ATCC 10556, and *Streptococcus salivarius* ATCC 13419 was evaluated using an Agar disc diffusion and minimum inhibitory concentration methods. Five formulations of the experimental gel were prepared at 0.25%, 0.5%, 1%, 1.5%, and 2% (v/v). The antibacterial susceptibility test was evaluated using an Agar‐Well diffusion assay against a bacterial consortium of *S. mutans*, *S. sanguinis*, and *S. salivarius*. The physical properties, pH, spreadability, gel morphology, phase separation, and drug release were evaluated. The experimental gels were compared with a chlorhexidine gel. Data were analyzed with one‐way ANOVA and Kruskal–Wallis tests with a significant level of 5%.

**Results:**

The major components of the EO were bornyl acetate, α‐isomethyl‐*E*‐nerolidol, germacrene B, *E*‐nerolidol, α‐cedrene‐epoxide, fokienol, and 10‐epi‐γ‐eudesmol. All formulations were effective in inhibiting bacterial growth. The 2% concentration presented inhibition zones (18.14 ± 1.01 mm) similar to those observed for the chlorhexidine gel (*p* > 0.05). All formulations were stable, without signs of separation, with adequate physical properties, and no significant differences were observed regarding the drug content with the chlorhexidine gel (*p* > 0.05).

**Conclusions:**

The experimental gels based on *G. tarapacana* EO presented good physicochemical properties and were highly effective in inhibiting the growth of a cariogenic bacterial consortium.

## 1 Introduction

Dental caries is a highly prevalent oral condition, impacting over 80% of the adult population and approximately 50% of the child population (Kazeminia et al. 2020). Dental caries is the product of dysbiosis caused by the proliferation of acidogenic and aciduric microbiota due to external factors, such as the high consumption of carbohydrates and sugars (Rosier, Marsh, and Mira 2018).

*Streptococcus mutans* is considered the main cariogenic bacteria in the oral cavity and has the potential to produce glucan‐binding polymerases that allow bacterial adhesion to tooth enamel, promoting a niche for the colonization and establishment of the insoluble matrix of dental plaque (Zhu et al. 2023). *Streptococcus sanguinis* is an early colonizer of dental plaque, and its ability to produce hydrogen peroxide can alter the pH, influencing the development of other species; thus, when the pH is reduced, the development of acid‐fast bacteria such as *S. mutans* and *Lactobacillus* is facilitated (Vaknin et al. 2020 *)*. *S. salivarius* is a commensal bacterium and an early colonizer of the oral microbiota. *S. salivarius* can influence the colonization and growth of other bacteria in a mixed biofilm. Furthermore, it has been isolated from areas with deep carious lesions. Therefore, its presence indicates cariogenic activity *(*Gross et al. 2012; Begić et al. 2023).

Traditional caries treatment includes physical removal of the lesion and restitution of the lost tissue with biomaterials. New strategies are being developed to promote disease prevention by taking a conservative approach to dental tissue. There are several options for preventing cavities, including maintaining good oral hygiene, using topical antimicrobials, undergoing fluoride therapy, and applying pit and fissure sealants (Lee 2013).

The development of preventive interventions can lead to positive changes in oral health practices (Veiga et al. 2023). Thus, preventive management can focus on preventing the proliferation of the central cariogenic microbiota and the formation of biofilms (Zhu et al. 2023). Natural products have been used as valuable resources for the development of therapeutic agents for various diseases, including the control of dental caries, mainly through the inhibition of bacterial growth (Jeon et al. 2011). Plant‐derived essential oils are a complex mixture of diverse components, mainly derived from terpenoids and aromatic and aliphatic constituents (Freires et al. 2015; Bakkali et al. 2008; Dagli et al. 2015). Some studies have compared the use of dental products such as toothpastes and gels for the control and inhibition of the growth of cariogenic microorganisms (Karadağlıoğlu et al. 2019; Badekova et al. 2021; Oluwasina et al. 2023; de Oliveira Carvalho et al. 2020; Piekarz et al. 2017). de Oliveira Carvalho et al. demonstrated that toothpastes formulated with essential oils of cinnamon, rosemary, nutmeg, and orange were effective in inhibiting the growth of *S. mutans*, *Enterococcus faecalis*, *S. aureus*, and *Lactobacillus lactis* and that the main constituents of the oils were phenolic compounds such as cinnamaldehyde, eugenol, thymol, and carvacrol. Recently, the essential oil of *Hypericum laricifolium* Juss has been used in the formulation of an experimental toothpaste, demonstrating significant inhibitory activity against a bacterial consortium formed by *S. mutans, S. sanguinis, and S. salivarius*. The main constituents of this essential oil were *n*‐octane and α‐pinene (Sánchez‐Tito et al. 2024).

*Grindelia tarapacana* is a species endemic to southern Peru and the northern desert of Chile (Granda, Bartoli, and Tortosa 2000). *G. tarapacana* belongs to the Asteraceae family and is a resinous perennial shrub with leathery leaves and yellow flowers. *G. tarapacana* has the particularity of producing a hydrophobic, non‐volatile resin on the surface of the plant as part of a cuticular layer (Wollenweber et al. 1993). Numerous diterpenoids have been identified in *G. tarapacana* samples (Zhou et al. 1995). Diterpenoids are secondary metabolites that have been shown to have important antibacterial activity (Saha et al. 2022), making them candidates for the development of new pharmacological formulations. Thus, more research is needed to prevent dental caries formation based on an approach to control cariogenic microbiota. To our knowledge, there are no previous reports on the use of *G. tarapacana* derivatives as anticariogenic agents. Therefore, the present study aimed to formulate, characterize, and evaluate the antibacterial activity of an experimental gel with various concentrations of *G. tarapacana* essential oil in a cariogenic bacterial consortium.

## 2 Materials and Methods

### 2.1 Ethical Approval and Sample Size Calculation

This research was approved by the research ethics committee of the Faculty of Health Sciences at the Private University of Tacna with the registration FACSA‐CEI/020‐05‐2023. The sample size was calculated using the G*Power 3.1.3 software (Heinrich Heine Universität, Düsseldorf, Germany). A one‐way fixed‐effects ANOVA test was used. An effect size of 0.75 was determined based on the pooled standard deviation from a previous study (Randall, Seow, and Walsh 2015). An α error probability of 0.05 and a power of 0.8 were considered. A total of five repetitions per experimental gel and the control group were calculated (*n* = 5). The power achieved with this setup was 0.82.

### 2.2 Extraction and Preparation of Essential Oil

Aerial parts of *G. tarapacana* were collected from the Characato District, Arequipa Province, Arequipa, Peru, at an approximate altitude of 2459 m.a.s.l., with coordinates 16° 29’ 90 S; 71° 51′ 91” W. Extraction of the essential oil was carried out by hydrodistillation for 4 h using a Clevenger apparatus. The essential oil (EO) was dried with sodium sulfate and stored at −20°C. Solutions of the EO at 80%, 60%, 40%, 20%, 10%, 5%, and 2.5% were prepared using dimethyl sulfoxide (DMSO; Loba Chemie, Mumbai, India).

### 2.3 GC‐MS Analysis

The composition of the EO was determined using gas chromatography‐mass spectrometry (GC‐MS). Initially, 20 µL of the EO was diluted in 980 mL of acetone. One microliter of the diluted EO was then injected into a Shimadzu CGMS‐QP2010 gas chromatograph‐mass spectrometer, which features a quadrupole analyzer from the same brand. For the analysis, a Restek Rtx‐5MS column was used (30 m length x 0.25 mm internal diameter). The run conditions were set as follows: an initial oven temperature of 60°C held for 5 min, a final temperature of 280°C maintained for another 5 min, and a heating rate of 2°C/min. The total run time was 120 min, with a column flow rate of 1.48 mL/min and a split ratio of 1:5. The identification of compounds was conducted based on retention times, Kovats indices, and by comparing experimental mass spectra with those found in the WILEY 229 and NIST107 libraries and literature (Adams 2007).

### 2.4 Disk Diffusion Assay

For the antibacterial susceptibility test, strains of *Streptococcus mutans* ATCC 25175, *Streptococcus sanguinis* ATCC 10556, and *Streptococcus salivarius* ATCC 13419 were used. The strains were plated in Petri dishes containing Brain–Heart Agar and incubated at 37°C for 24 h. Subsequently, colonies of each strain were transferred to test tubes containing Brain–Heart infusion broth and incubated until they reached the exponential growth phase. The suspension of each bacterium was adjusted according to the McFarland 0.5 standard. Petri dishes containing Brain–Heart Agar were seeded with each strain using the swabbing method. Filter paper discs (Whatman; Merck KGaA, Darmstadt, Germany) with a diameter of 6 mm were loaded with 10 µL of each concentration of EO and placed on the plates. Petri dishes were incubated at 37°C for 24 h. The assay was performed in triplicate for each concentration and the bacterial strain. Culture media were purchased from Liofilchem (Abruzos, TE, Italy).

### 2.5 Determination of Minimum Inhibitory Concentration (MIC)

The MIC of the various concentrations of the EO was determined by the broth microdilution method (da Silva et al. 2020; Silva et al. 2021). Briefly, a 190 µL aliquot of a bacterial suspension of *S. mutans* was added into a 96‐well microplate. Then, a 10 µL aliquot of the EO at the adequate concentration was added to the corresponding wells. The microplates were incubated at 37°C for 24 h. Chlorhexidine at 0.12% (Perio.Aid, Dentaid, Barcelona, Spain) and DMSO were used as positive and negative controls, respectively. The inhibition of bacterial growth was assessed visually, and 2 µL from each well was transferred to a Petri dish containing Brain–Heart Agar and incubated at the same conditions. Subsequently, bacterial growth was evaluated by counting colony‐forming units (CFU). Due to the methodology employed, the final test concentration in the microdilution broth assay was diluted by a factor of 20. The assay was carried out for *S. sanguinis* and *S. salivarius* in triplicate.

### 2.6 Preparation of the Experimental Gel

The experimental base gel was prepared using the following procedure: A beaker containing 50 mL of distilled water was placed on a magnetic stirrer at 45°C and 400 rpm for 5 min. Then, 0.1 g of methylparaben, 0.05 g of sodium saccharin, and 0.05 g of menthol were slowly added, and the mixture was constantly stirred for 5 min. Next, 14 mL glycerol was slowly added. Finally, 1.25 g of Carboxymethyl cellulose was added in small quantities to ensure complete incorporation. The mixture was magnetically stirred for 30 min to ensure complete homogenization. All the reagents were purchased from ISDA EIRL (Lima, Peru).

Five formulations of the experimental gel were prepared based on *G. tarapacana* EO at 0.25, 0.5, 1, 1.5, and 2% (v/v). For this, 5 mL of the base gel was added to sterile syringes, and 62.5, 100, 250, 375, and 500 µL of the EO at 20% was added with a micropipette because this concentration proved to be effective in the disk diffusion assay and maintains an effective MIC to inhibit the growth of the three bacterial strains. Constant mixing was performed for 1 min to ensure complete homogenization. The volume of EO used ensured a concentration of 217.52, 163.14, 108.76, 54.38, and 27.19 mg/mL of the active agent in each experimental gel, respectively. For the control group, Perio.Aid dentifrice gel (Dentaid, Barcelona, Spain) was used.

### 2.7 Evaluation of Physical Properties

The physical properties were evaluated on days 0, 4, 8, and 12 to verify their consistency over time. The color of the gels was assessed visually; 3 mL of the gel was placed in a tube, and the color was checked against a black background. To evaluate the homogeneity of the formulations, a small amount of each gel was placed on a slide and covered with a coverslip. The mixture was allowed to rest for 5 min. Each slide was evaluated to demonstrate the homogeneous distribution of the gel without visible granulations (Harahap, Nainggolan, and Harahap 2018). The odor of the formulations was assessed after mixing 0.5 mL of gel in a tube containing 3 mL of distilled water, and mixing was carried out for 1 min in a vortex (Pawar et al. 2013). Consistency and greasiness were assessed by placing 100 mg of the drug directly on the skin.

### 2.8 Determination of the pH

The pH determination was carried out with a pH meter. 2.5 g of each gel was weighed and dispersed in 25 mL of distilled water. The solutions were then stored at room temperature for 2 h. The pH measurements were performed in triplicates (Rompicherla et al. 2022).

### 2.9 Gel Morphology

Ten milligrams of each formulation were placed on a slide and observed under a light microscope at 10× and 40× to evaluate the globular structure of the gel (Alam et al. 2023).

### 2.10 Determination of Spreadability

To determine spreadability, the method described by Rachit et al. (2011) was used, with some modifications. Briefly, 350 mg of each gel was placed on a (12 × 8 cm) glass plate. Another glass slide weighing 8.5 g was dropped from a distance of 5 mm and left for 1 min. Subsequently, the spreading circle of each gel was measured using a digital Vernier and compared with the classification of gel types based on the spreadability proposed by Dignesh, Ashish, and Dinesh (2012).

### 2.11 Determination of Separation Phase

The gel formulations were centrifuged. Each formulation (5 g) was placed in a 15 mL Falcom tube and centrifuged at 3500 rpm for 30 min (Khan et al. 2022). If the presence of a separation liquid phase was observed, the supernatant liquid was decanted and weighed to calculate the percentage of separation % (w/w) according to Equation 1 (Shahin, Hamed, and Alkhatib 2015):

(1) $$\mathrm{Separation}\;\%\;\left(w/w\right)=\frac{\mathrm{Weigth}\;\mathrm{of}\;\mathrm{separated}\;\mathrm{liquid}\;\mathrm{phase}}{\mathrm{Initial}\;\mathrm{weight}\;\mathrm{of}\;\mathrm{sample}}\;\times \;100$$

### 2.12 Determination of Drug Content

The λ max of a 20% essential oil solution was determined using a UV–vis spectrophotometer in the 190–450 nm range. The absorbance detected at a λ max of 416 nm was 0.041, this was considered as the maximum absorbance of the standard (Ali Khan et al. 2020). The determination of the drug content of the formulations was carried out following the recommendations of Khan et al. (2022). Briefly, 1 g of each gel was dissolved in 10 mL of 80% ethanol, made up with ethanol to a volume of 100 mL. The absorbance was calculated for the essential oil formulations and chlorhexidine at a λmax of 416 nm. The drug concentration was calculated according to Equation 2 (Khan et al. 2022):

(2) $$\%\;\mathrm{of}\;\mathrm{drug}\;\mathrm{concentration}=\frac{\mathrm{Absorbance}\;\mathrm{of}\;\mathrm{sample}}{\mathrm{Absorbance}\;\mathrm{of}\;\mathrm{standard}}\;\times 100$$

### 2.13 Agar‐Well Diffusion Assay

To evaluate the antibacterial susceptibility of the gels, a bacterial consortium was prepared using *S. mutans* ATCC 25175, *S. sanguinis* ATCC 10556, and *S. salivarius* ATCC 13419. An aliquot from the activated suspensions of the strains was transferred to a tube containing Brain–Heart infusion broth and incubated at 37°C for 24 h. Subsequently, a suspension adjusted to the McFarland scale (0.5) was prepared and seeded in Petri dishes containing Brain–Heart Agar that were allowed to rest for 10 min. Next, with a biopsy punch, 6 mm wells were made on the surface of the agar. Approximately 100 mg of each gel was added to each well (Karadağlıoğlu et al. 2019). Petri dishes were incubated at 37°C for 24 h under microaerophilic conditions. For the control group, Perio. Aid® dentifrice gel (Dentaid, Barcelona, Spain) was used. The formation of inhibition zones was measured using a digital Vernier. Five repetitions were performed for each gel sample.

### 2.14 Statistical Analyses

Stata software version 17 (StataCorp LP, College Station, TX, USA) was used for the statistical analysis. To verify the differences between the bacterial growth inhibition values of the toothpaste, the one‐way analysis of variance was chosen after checking the assumptions of independence, and at the level of the residuals, the normal distribution was evaluated with the Shapiro–Wilk test; for the homogeneity of variances, the Breusch–Pagan/Cook–Weisberg test was used, and to verify if the mean of the residuals was equal to zero, a one‐sample *t*‐test was performed. To evaluate the physicochemical properties, the Kruskal–Wallis test was used. The Bonferroni post‐hoc test was used to identify differences between pairs of groups. In addition, the effect size was evaluated using the eta‐squared estimator. Graphs were constructed using GraphPad Prism version 10.0.0 (GraphPad Software, Boston, Massachusetts, USA). A significance level of 5% was set for all the tests.

## 3 Results

### 3.1 GC–MS Analysis

Chromatographic analysis of the volatile oils from the aerial parts of *G. tarapacana* showed bornyl acetate, α‐isomethyl‐*E*‐nerolidol, germacrene B, *E*‐nerolidol, α‐cedrene‐epoxide, fokienol, and 10‐epi‐γ‐eudesmol as major components (Table 1).

**Table 1:** Essential oil composition of *G. tarapacana*.

N°	Compound	RT	KI	%
1	Bornyl acetate	49.83	1288	5.14
2	α‐isomethyl‐*E*‐nerolidol	58.97	1479	4.94
3	Germacrene B	62.34	1561	5.68
4	*E*‐Nerolidol	62.75	1563	5.81
5	α‐Cedrene‐epoxide	63.13	1575	4.33
6	Fokienol	64.08	1596	6.21
7	10‐*epi*‐γ‐Eudesmol	65.33	1623	5.30
Oxigenated monoterpene				5.14
Sesquiterpene hydrocarbon				5.68
Oxigenated Sesquiterpene				26.59

Abbreviations: RT, retention time; KI, Kovats indices.

### 3.2 Antibacterial Activity of *G. tarapacana* Essential Oil

**Figure 1:**
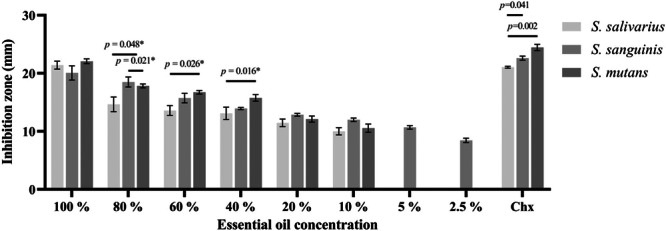
Antibacterial activity of *G. tarapacana* essential oil against *S. mutans*, *S. sanguinis*, and *S. salivarius* (inhibition zones in millimeters). The values of the individual experiments are expressed as the mean ± standard error of three independent experiments. *p < 0.05.

In the agar disk diffusion assay, *G. tarapacana* EO showed moderate to strong inhibition of bacterial growth of *S. mutans*, *S. sanguinis*, and *S. salivarius* at concentrations greater than 10%. No statistically significant differences were observed at the 100% and 20% concentrations among the three strains (*p* > 0.05). *G. tarapacana* EO was more effective at 80%, 60%, and 40% concentrations for *S. mutans* than for *S. salivarius* (*p* < 0.05). Concentrations less than 10% will not be considered effective in inhibiting the growth of bacterial strains, as the inhibition zones will not exceed 12 mm. Chlorhexidine was effective in inhibiting the growth of all three strains. It was more effective against *S. mutans* (*p* < 0.05) (Figure 1).

In the MIC test, the different concentrations of EO showed strong inhibition against *S. mutans*, *S. sanguinis*, and *S. salivarius*. *G. tarapacana* EO showed complete inhibition of the growth of *S. mutans* at a concentration of 5% (MIC 2.74 mg/mL), 10% (MIC 5.44 mg/mL) for *S. sanguinis*, and 2.5% (MIC 1.35 mg/mL) for *S. salivarius* (Table 2).

**Table 2:** Minimum Inhibitory Concentration (MIC) of *G. tarapacana* essential oil against *S. mutans*, *S. sanguinis,* and *S. salivarius*.

Concentration % (mg/mL)	MIC (mg/mL)	*S. mutans* (CFU)	*S. sanguinis* (CFU)	*S. salivarius* (CFU)
100% (1087.60)	54.38	< 3	< 3	< 3
80% (870.04)	43.50	< 3	< 3	< 3
60% (652.56)	32.62	< 3	< 3	< 3
40% (435.04)	21.75	< 3	< 3	< 3
20% (217.52)	10.87	< 3	< 3	< 3
10% (108.76)	5.44	< 3	< 3	< 3
5% (54.38)	2.72	< 3	242	< 3
2.5% (27.19)	1.35	> 300	> 300	< 3
CHX 0.12%	—	< 3	< 3	< 3
DMSO	—	> 300	> 300	> 300

Abbreviations: CFU, colony‐forming unit; MIC, minimum inhibitory concentration.

### 3.3 Physicochemical Evaluation

The gel formulations were evaluated physically and visually for color, odor, homogeneity, consistency, and greasiness (Table 3). In general, no major variations were observed in the properties of the formulations. It was observed that as the concentration of EO increased, the color varied and became opaque, whereas the consistency, homogeneity, and greasiness of the gels were shown to be good, excellent, and non‐greasy, respectively. Regarding odor, all gels were aromatic, presenting a spicy herbal aroma based on *G. tarapacana* and peppermint for chlorhexidine.

**Table 3:** Physical characteristics of the gels.

Gel formulation	Color	Homogeneity	Odor	Consistency	Greasiness
F1 (0.25%)	Light opaque white	Good	Spicy herbal	Good	Nongreasy
F2 (0.5%)	Opaque white	Good	Spicy herbal	Good	Nongreasy
F3 (1%)	Opaque white	Excellent	Spicy herbal	Good	Nongreasy
F4 (1.5%)	Opaque white	Excellent	Spicy herbal	Good	Nongreasy
F5 (2%)	Opaque white	Excellent	Spicy herbal	Good	Nongreasy
F6 (Chx)	Turquoise green	Excellent	Peppermint	Good	Nongreasy

The experimental gels showed pH values of 7.02–7.17, close to oral pH. All formulations were shown to be stable, showing no signs of phase separation, and were considered fluid gels according to Dignesh, Ashish, and Dinesh (2012). When evaluating spreadability, no significant differences were observed between the formulations, except for the 0.5% gel and chlorhexidine (*p* < 0.05) (Table 4). The drug contents of the formulations ranged from 94.31% to 104.06%, with no significant differences compared to the drug content for the chlorhexidine gel (*p* > 0.05) (Table 4).

**Table 4:** pH, Spreadability, separation percent, and drug content of the formulated gels.

Gel formulation	pH* Mean ± SD	Spreadability (mm)* Mean ± SD	Separation percent (%)	Drug content* (%)
F1 (0.25%)	7.02 ± 0.75^a^	22.40 ± 0.24^a^	Nil	94.31 ± 2.82^a^
F2 (0.5%)	7.02 ± 0.11^a^	23.21 ± 0.44^ab^	Nil	95.93 ± 1.41^a^
F3 (1%)	7.11 ± 0.40^a^	22.34 ± 0.14^ab^	Nil	97.56 ± 2.44^a^
F4 (1.5%)	7.16 ± 0.52^a^	22.52 ± 0.04^ab^	Nil	100.81 ± 3.72^a^
F5 (2%)	7.18 ± 0.10^a^	23.30 ± 0.18^ab^	Nil	104.06 ± 1.41^a^
F6 (Chx)	7.17 ± 0.03^a^	23.63 ± 0.08^b^	Nil	111.67 + 3.82^a^

* Kruskal–Wallis test followed by the Dunn test. The same superscript letters indicate no statistically significant differences between the groups (*p* > 0.05).

### 3.4 Antibacterial Activity of the Gel Formulations

The experimental gel formulations based on *G. tarapacana* EO showed antibacterial activity against the bacterial consortium. Susceptibility was calculated in terms of bacterial growth inhibition zones, and significant differences were observed among all formulations (*p* < 0.05). As the concentration (% v/v) of EO as the active product in the gel matrix increased, the inhibition zones were greater. No statistically significant differences were observed between the antibacterial susceptibility at concentrations of 1%, 1.5%, 1.5%, and chlorhexidine (*p* > 0.05). Concentrations greater than 1% generated inhibition zones of greater than 13 mm. The 2% concentration presented inhibition zones of 18.14 ± 1.01 mm, greater than those of chlorhexidine (16.95 ± 0.43 mm), and these differences were nonsignificant (*p* > 0.05). The estimated effect size (*η* ^2^ = 0.956) indicates that 95.6% of the variability in susceptibility against the bacterial consortium is attributable to the different concentrations of *G. tarapacana* EO contained in the gel (Table 5).

**Table 5:** Antibacterial activity of the *G. tarapacana* gels and Chlorhexidine against the bacterial consortium (inhibition zones in mm).

Gel Formulation	Mean ± SD	Min	Max	*p*‐value*	*η* ^2^
F1 (0.25%)	8.10 ± 0.60	7.17	8.67	< 0.001	0.956
F2 (0.5%)	10.28 ± 0.81	9.46	11.48		
F3 (1%)	14.77 ± 1.28^a^	13.31	15.96		
F4 (1.5%)	16.10 ± 0.78^ab^	15.16	17.03		
F5 (2%)	18.14 ± 1.01^c^	17.01	19.56		
F6 (Chx)	16.95 ± 0.43^bc^	16.27	17.48		

* One‐way ANOVA followed by the Bonferroni test. The same superscript letters indicate no statistically significant differences between the groups (*p* > 0.05). SD, Standard deviation; *η* ^2^, Eta squared.

## 4 Discussion

Experimental formulations of toothpastes and gels have been proposed to inhibit the growth of microorganisms associated with caries formation; most of these reports have been carried out in vitro studies. Generally, the inclusion of extracts and essential oils from various plants have been incorporated as active agents, recognizing their important antimicrobial properties (Karadağlıoğlu et al. 2019; Badekova et al. 2021; Oluwasina et al. 2023; de Oliveira Carvalho et al. 2020; Piekarz et al. 2017; Sánchez‐Tito et al. 2024). Therefore, the search for new therapeutic options based on natural products that offer better clinical benefits than conventional preventive treatments has been recommended (Arena et al. 2022).

The EO tested in this study has not been previously used for the formulation of products for dental use; its antibacterial activity indicates its potential use as an active agent in the synthesis of new products to control caries formation. The EO of *G. tarapacana* was compared against chlorhexidine, the results showed that concentrations greater than 20% were effective against *S. mutans*, *S. sanguinis*, and *S. salivarius*, although only the 100% EO obtained inhibition zones similar to those of chlorhexidine. The results showed that the experimental gels based on *G. tarapacana* essential oil had an important antibacterial activity against microorganisms associated with dental caries such as *S. mutans*, *S. sanguinis*, and *S. salivarius*.

The evaluation of physicochemical properties is a relevant aspect of the development of pharmacological formulations (Liu, Müller, and Möschwitzer 2015). Appropriate characteristics of color, odor, homogeneity, consistency, and fattiness can facilitate product acceptance. In this regard, in this study, all gel formulations based on *G. tarapacana* EO presented similar physicochemical properties, even with the commercial chlorhexidine gel. Only the spicy herbal aroma of the experimental gels turned out to be very invasive, unlike the menthol aroma of the chlorhexidine gel. Microscopic evaluation of the gel showed the formation of spherical globules. According to Naga et al., its presence is indicative of the gel emulsion formulation (Naga Sravan Kumar Varma et al. 2014).

Spreadability is one of the most important aspects for the evaluation of formulations, this can affect the effectiveness of the product, it depends on other properties such as viscosity and the properties of the polymers contained in the gel matrix (Rompicherla et al. 2022; Khan et al. 2022). In this study, it was found that the spreadability was adequate with values between 22.4 and 23.63 mm. The values indicate that the gels can be easily expandable with light pressure and can be considered as fluid gels (Dignesh, Ashish, and Dinesh 2012). It was observed that the experimental gels presented pH values close to those of the oral environment. Evaluation of drug content suggests a uniform distribution for each concentration.

The *G. tarapacana* EO at a concentration of 20% contains 217.52 mg/mL of active agent, which is because it was used to prepare the experimental gels. Although there are no reports in the literature on the antibacterial activity of gels based on *G. tarapacana*, which makes comparisons difficult, some close studies have evaluated the properties of pastes and gels containing extracts and essential oils of various medicinal plants. A study evaluated the antibacterial activity of toothpaste with various plant extracts from India. The results showed that the toothpaste was effective against Gram‐positive and Gram‐negative bacteria (Parveen et al. 2021).

Pawar et al. (2013). demonstrated that the formulation of a dental gel based on coriander EO was effective in inhibiting the growth of *S. mutans* (23 ± 2.6 mm), *S. sanguinis* (18 ± 4.2 mm), and *L. acidophilus* (18 ± 1.9 mm); Furthermore, the inhibition zones of the gel were similar to those of crude oil, which suggests that the incorporation of the active agent in the base of the gel does not decrease its antibacterial activity. Another study found that the inclusion of *Origanum vulgare* extract and EO in a dental gel generated inhibition zones of greater than 20 mm for *Staphylococcus aureus*, *Bacillus subtilis*, *Escherichia coli*, and *Candida albicans (*Badekova et al. 2021 *)*. Nuraskin et al. (2021). demonstrated that a toothpaste based on Laban leaf extract showed a minimum inhibitory concentration against *S. mutans* at a concentration of 4.5% with an average number of bacteria of 108.5 × 10^−7^ CFU/mL. All these studies highlight the important antibacterial activity of derivatives of natural products on microorganisms associated with the formation of dental caries. In the present study, gels containing low concentrations of *G. tarapacana* EO showed antibacterial activity against the bacterial consortium. The concentrations of 1.5% and 2% presented inhibition zones of 16.10 ± 0.78 mm and 18.14 ± 1.01 mm, respectively. This susceptibility was similar to that observed with the chlorhexidine gel (16.95 ± 0.43 mm).

The antibacterial activity of EOs is directly related to their composition, being a variable mixture of bioactive compounds, mainly terpenoids. The EOs of other species of the Grindelia genus have been shown to have antibacterial properties against various microorganisms such as *Streptococcus*, *Staphylococcus aureus*, and *C. albicans*; monoterpenoids being the main constituents (Poudel et al. 2023; Nowak et al. 2019). Zouh et al. (1995). identified the presence of labdane‐type diterpenes isolated from *G. tarapacana* from northern Chile; these diterpenes were tarapacol diacetate, tarapacol, tarapacol 15‐acetate, tarapacanol A 14,15‐diacetate, tarapacanil A, tarapacanone, tarapacanol B, as well as 13‐epimanoyloxide and 12 alpha‐hydroxy‐13‐epi‐mannoyloxide.

There are no previous studies that characterize the chemical components of *G. tarapacana* from the high Andean area of Peru. Thus, the major components of the *G. tarapacana* EO in this study were bornyl acetate, α‐isomethyl‐E‐nerolidol, germacrene B, E‐nerolidol, α‐cedrene‐epoxide, fokienol, and 10‐epi‐γ‐eudesmol. These compounds are mainly sesquiterpenes that have been shown to have significant anti‐inflammatory and antibacterial activity (Zhao, Sun, and Ruan 2023; Li et al. 2022).

The antibacterial action mechanism of EOs can vary according to their components, their characteristics, and whether the bacteria are Gram‐positive or Gram‐negative. One of the main characteristics of EOs is hydrophobicity, which allows them to divide with the lipids present in the cell membrane, making them more permeable by altering the cellular structure (Chouhan, Sharma, and Guleria 2017). In general, it has been proposed that the main mechanism of action is related to the irreversible damage of the bacterial cell wall and membrane, which leads to the leakage of proteins and DNA and RNA molecules, causing cell death (Valdivieso‐Ugarte et al. 2019; Meng et al. 2016). Particularly, it has been discussed that the mechanism of action of sesquiterpenoids is associated with the ability to destabilize the microbial cell membrane, due to a lipophilic activity on the cell wall (Ivanescu, Miron, and Corciova 2015; de Moura et al. 2021).

It is known that Gram‐positive bacteria are more susceptible to EOs since they have a thick peptidoglycan wall, which facilitates the access of antimicrobial molecules to the cell membrane (Zinoviadou, Koutsoumanis, and Biliaderis 2009). Furthermore, Gram‐positive bacteria can facilitate the infiltration of hydrophobic EO compounds due to the lipophilic ends of lipoteichoic acid present in the cell membrane (Cox et al. 2000). On the other hand, Gram‐negative bacteria have a rigid and more complex outer membrane, rich in lipopolysaccharides, which limits the diffusion of hydrophobic compounds. Urzúa et al. (2008) demonstrated that various diterpenoids derived from different plant species were effective against Gram‐positive bacteria but not against Gram‐negative bacteria. The mechanism of action of diterpenoids is associated with their ability to cross or damage bacterial cell membranes. Their results through a phospholipid bilayer model suggest that they have two structural characteristics for antibacterial activity: a substituted decalinic system, capable of inserting into a lipophilic region, and a hydrophilic fragment that possesses a hydrogen bond donor group, capable of having hydrogen bond acceptor groups in the membrane.

In this study, strains of *S. mutans*, *S. sanguinis*, and *S. salivarius* were used as a bacterial consortium to test the susceptibility of the experimental gels, considering that interactions can occur between these strains. They are Gram‐positive bacteria that have a thick cell wall, composed of peptidoglycans and teichoic acids that prevent osmotic lysis of the wall and confer rigidity and shape to the cell (Zhu et al. 2018, 2006; Chastanet and Msadek 2003). In most Gram‐positive oral bacteria, quorum sensing is coordinated by peptide pheromones, which act as extracellular signaling molecules to communicate biofilm formation (Valdivieso‐Ugarte et al. 2019). In this regard, it is known that essential oils can inhibit biofilm formation through the inhibition of communication of bacterial cells (Valdivieso‐Ugarte et al. 2019), this could explain the high susceptibility reported in this study.

## 5 Conclusions

Within the limitations of this study, it was found that the experimental gels based on *G. tarapacana* EO presented good physicochemical properties and were highly effective in inhibiting the growth of a bacterial consortium formed by *S. mutans*, *S. sanguinis*, and *S. salivarius*. The gels with a concentration of 1.5% and 2% v/v had a behavior similar to a commercial gel based on chlorhexidine. Alongside these promising findings, future in vitro and in vivo investigations are necessary to study other rheological properties, stability over time, and potential toxicity of the formulations.

## Author Contributions

**Marco Sánchez‐Tito:** conceptualization, methodology, software, formal analysis, investigation, resources, writing original draft preparation, writing, review and editing, visualization, project administration, funding acquisition. **Lidia Yileng Tay:** methodology, validation, writing, review, and editing. **Francisco Zea‐Gamboa:** methodology, formal analysis, data curation, supervision. **Raúl Cartagena‐Cutipa:** validation, formal analysis, supervision. **Alysson Flores‐Gómez:** investigation. **Bruno \Spigno‐Paco:** investigation, data curation. **Brando Raul Mendoza Salinas:** formal analysis. **Jose Elias Zuñiga Calcina:** formal analysis. **Ingrit Elida Collantes Díaz:** conceptualization, validation, writing review and editing, supervision. All authors gave their final approval and agreed to be accountable for all aspects of the work.

## Ethics Statement

This study was approved by the research ethics committee of the Faculty of Health Sciences at the Private University of Tacna, Peru (ref: FACSA‐CEI/020‐05‐2023).

## Conflicts of Interest

The authors declare no conflicts of interest.

## Data Availability

The data that support the findings of this study are available from the corresponding author upon reasonable request.
